# The importance of assessing left ventricular longitudinal function in presence of increased afterload

**DOI:** 10.1186/s13054-024-04801-w

**Published:** 2024-01-12

**Authors:** Cristina Santonocito, Ryota Sato, Siddharth Dugar, Filippo Sanfilippo

**Affiliations:** 1 Department of Anaesthesia and Intensive Care, A.O.U. Policlinico-San Marco, Site “Policlinico G. Rodolico”, Via S. Sofia N 78, 95123 Catania, Italy; 2 Division of Critical Care Medicine, Department of Medicine, The Queen’s Medical Center, Honululu, HI USA; 3 Department of Critical Care Medicine, Respiratory Institute, Cleveland Clinic, Cleveland, OH USA; 4 Cleveland Clinic Lerner College of Medicine, Cleveland, OH USA; 5 Department of General Surgery and Medical-Surgical Specialties, Section of Anesthesia and Intensive Care, University of Catania, Catania, Italy

Dear Editor,

We read with interest the findings reported by *de Courson *et al.[1] regarding cardiac dysfunction in patients with good-grade subarachnoid haemorrhage (SAH), which is associated with a potentially massive surge in catecholamines. The precise prevalence of cardiac dysfunction in this population remains unclear. A recent meta-analysis[2] of 23 studies focusing on SAH patients revealed large heterogeneity in clinical outcomes and suggested that about one in five patients with SAH develops cardiac dysfunction, similar to the prevalence of cardiomyopathy observed in sepsis[3]. Such an event seemed associated with higher in-hospital mortality [odds ratio 2.69 (1.64 to 4.41); *p* < 0.001; *I*^2^ = 63%], though with very low certainty of evidence. Most included studies used unspecific criteria for diagnosis of cardiac dysfunction such as regional wall motion abnormality, while only three studies used left ventricular ejection fraction (LVEF), and the other two defined cardiac dysfunction by speckle‑tracking echocardiography.

Hence, *de Courson *et al.[1] should be commended for performing an advanced and detailed study using speckle‑tracking echocardiography with global longitudinal strain (GLS) analysis. Importantly, the authors investigated the relationship between GLS and LVEF. We think that a couple of aspects deserve comments and more in-depth analysis.

First, the authors reported a very high prevalence of cardiac dysfunction (60.6%) when using GLS − 20% as a cut-off, but this prevalence decreased to 21.2% when using a more restrictive cut-off for cardiac dysfunction (GLS − 17%). We believe that the latter cut-off is reasonable. For instance, a recent meta-analysis showed that longitudinal strain in septic patients averages between − 14 and − 15%, with a 1.45% absolute mean difference according to survival[4], similarly to findings previously published in the journal[5]. Such findings reinforce the belief that GLS cut-off used in the outpatient population may not be appropriate for critically ill patients.

Second, the authors confirmed that LVEF may not be a valuable parameter to identify early cardiac dysfunction in this population, being altered in only 1.7% and showing no correlation with GLS (*p* = 0.69). This may be explained by an alteration in myocardial longitudinal deformation (GLS) at earlier stages as compared to LVEF. This is not surprising as an initial reduction in longitudinal contractility may be compensated by an increased circumferential function so that LVEF results are just marginally affected as opposed to GLS at earlier stages. Moreover, GLS is very sensitive to afterload[6–10]. For instance, in patients with aortic stenosis GLS is significantly more sensitive to the variation of afterload than LVEF[9, 10]. Considering the potentially massive catecholamine release in patients with SAH[11], systemic vascular resistances may increase substantially in these patients and LVEF may not be a valuable variable. In the study by *de Courson *et al.[1], both mean arterial pressure and systemic vascular resistances were high (ranges of median 100 mmHg and 1606–1866 dyn·sec·cm^−5^, respectively), which may have led to an alteration of GLS as a sign of subclinical LV systolic dysfunction. In this regard, the authors reported a third parameter investigating cardiac contractility, the mitral s’ wave obtained with tissue Doppler imaging. This parameter investigates myocardial longitudinal function and, for this reason, may be a better surrogate of GLS as compared to LVEF. Our group performed a large study in septic showing that mitral s’ and LVEF were not interchangeable, with only a moderate correlation[12]. We would ask the authors to analyse and report on the correlation and concordance of mitral s’ and GLS in patients with SAH. Considering the much higher availability and greater feasibility of tissue Doppler imaging measurements as compared to the limited feasibility of obtaining strain imaging (available in only 43% of enrolled patients) in intensive care[13], it would be valuable for clinicians to understand whether mitral s’ wave could be used as a surrogate of GLS.

## Data Availability

Not applicable.
